# Age-Related Change in Visual Working Memory: A Study of 55,753 Participants Aged 8–75

**DOI:** 10.3389/fpsyg.2013.00012

**Published:** 2013-01-29

**Authors:** James R. Brockmole, Robert H. Logie

**Affiliations:** ^1^Department of Psychology, University of Notre DameNotre Dame, IN, USA; ^2^Philosophy, Psychology, and Language Science, University of EdinburghEdinburgh, UK

**Keywords:** visual working memory, ageing, binding, objects, internet

## Abstract

Visual working memory (VWM) abilities of 55,753 individuals between the ages of 8 and 75 were assessed to provide the most fine-grain analysis of age-related change in VWM to date. Results showed that VWM changes throughout the lifespan, peaking at age 20. A sharp linear decline follows that is so severe that by age 55, adults possess poorer immediate visual memory than 8 and 9 year olds. These developmental changes were largely explained by changing VWM capacity coupled with small short-term visual feature binding difficulties among children and older adults.

## Introduction

Visual objects are defined by a range of basic visual features such as color, shape, luminance, size, orientation, and texture. Therefore, accurate recall of objects depends not only on the amount of information that can be retained in memory, but also on each individual’s ability to bind or properly associate these various features in memory. Recent investigations have considered whether this binding ability, in addition to memory capacity, varies as a function of age. For example, one’s ability to remember object location bindings in visual working memory (VWM)^1^ appears to be susceptible to the aging process. Both children and senior citizens seem to have more difficulty maintaining memory for object location than do young adults in their twenties (e.g., Mitchell et al., 2000; Cowan et al., 2006). This inverted-*U* shaped trend in binding ability perhaps parallels growth and decline in several memory-based abilities in childhood and old age (e.g., Logie and Maylor, 2009; Maylor and Logie, 2010).

Age-related binding deficits, however, may not be universal. After reaching adulthood, bindings between surface features such as color and shape seem to be generally unaffected by normal aging (Brockmole et al., 2008; Parra et al., 2009; Brown and Brockmole, 2010). This result is somewhat surprising given that age-related feature-based binding deficits exist in long-term memory (Chalfonte and Johnson, 1996; Naveh-Benjamin, 2000; Naveh-Benjamin et al., 2004). Given this contrast, at least one recent result suggesting an age-related surface-feature binding deficit in VWM (Brown and Brockmole, 2010), and the relative lack of data on such binding abilities in childhood, the present study revisits the issue with a sample size large enough to detect even very small age-related changes in binding abilities from childhood to very old age.

## Materials and Methods

Data were collected from 160,405 volunteer participants who spontaneously accessed the Science pages on the British Broadcasting Corporation (BBC) website between May 2006 and March 2007 and completed a set of tests of different aspects of memory^2^. Here, we focus on results from the test of visual feature binding from this set. Results from the other tests in the set, and additional data from the same source, are reported elsewhere (e.g., Logie and Maylor, 2009; Johnson et al., 2010; Maylor and Logie, 2010). Participants reported 138 different countries of residence, but the vast majority reported residing in the United Kingdom or United States. Initial data analysis revealed no differences between those reporting residence in English language-dominated countries and those that did not. Although participants were not specifically asked to indicate fluency in English, we considered it reasonable to assume that they had a high level of English fluency because they had to find and to choose to access the study through English language web pages maintained by the BBC.

**Figure 1:**
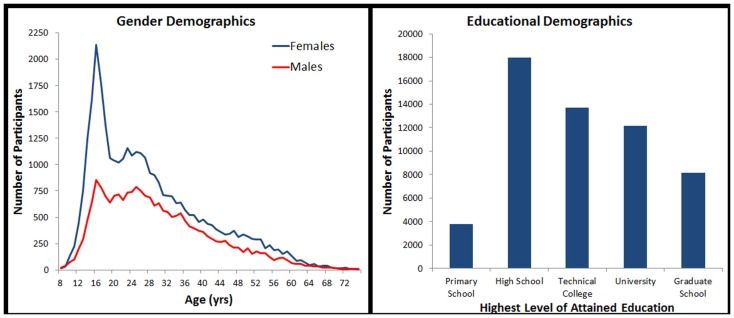
**Sample demographics broken down by age, gender, and education**.

For this study, we made use of a subset of the data records collected. We excluded all those under the age of 8, over the age of 75, and those who did not provide age, gender, education, country of residence, or a general health rating of “Good” or “Excellent.” In an effort to include only serious attempts to complete the task, we additionally excluded participants who did not provide at least one correct response. Finally, because it was impossible to ascertain how many times a particular individual completed the tests, we made use of only the first data record from each computer in order to minimize the frequency of repeated attempts in the data set(cf. Logie and Maylor, 2009; Johnson et al., 2010). This left a total of 55,753 data records for analysis (see Figure 1).

**Figure 2:**
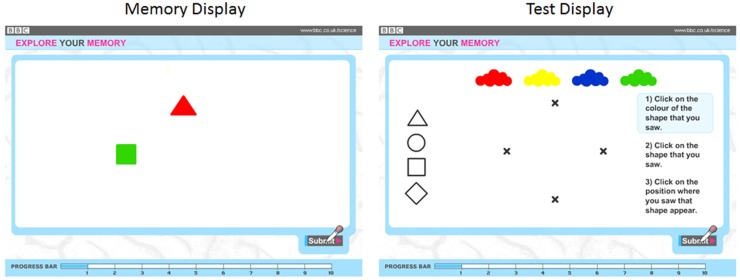
**Sample memory (left) and test (right) displays used during the experiment**. In this example, the observer is to remember the color, shape, and location of two objects.

Observers were presented with displays containing one to four colored objects(an example trial is illustrated in Figure 2). Approximately half of the observers (28,171) were shown geometric shapes (circle, square, triangle, diamond) with the remaining observers (27,582) shown line drawings of animals (camel, penguin, elephant, pig). Preliminary analyses showed that there were no reliable differences in the data across the two stimulus types, and analyses were collapsed across this design factor. Objects were red, yellow, green, and blue. Colors and shapes were combined without replacement. Displays of 1, 2, 3, and 4 objects were shown for 2, 4, 6, and 8 s, respectively.

Following object offset, four color patches and four shape outlines appeared along the top and left edges of the screen respectively. All four possible object locations were marked with *x*’s. Observers recalled objects by clicking on a color patch, then clicking on a shape, and then clicking on a location. Observers were shown up to two trials per set size, beginning with a set size of 1, and progressively increasing. The test stopped if a participant failed to recall all three features for all the objects in the study display on two successive trials. Performance was scored as the number of objects for which color, shape, and location were reported correctly and thus ranged from 1 to 20. While additional trials would have allowed finer grain measurement of memory both within and across individuals, the difficulty, and duration of the task were of greater concern in a set of tests that were offered online for the purpose of both research and community engagement in science. This level of task demand helped ensure task completion by a substantial number of individuals, and the resulting number of participants yielded substantial statistical power.

## Results

**Figure 3:**
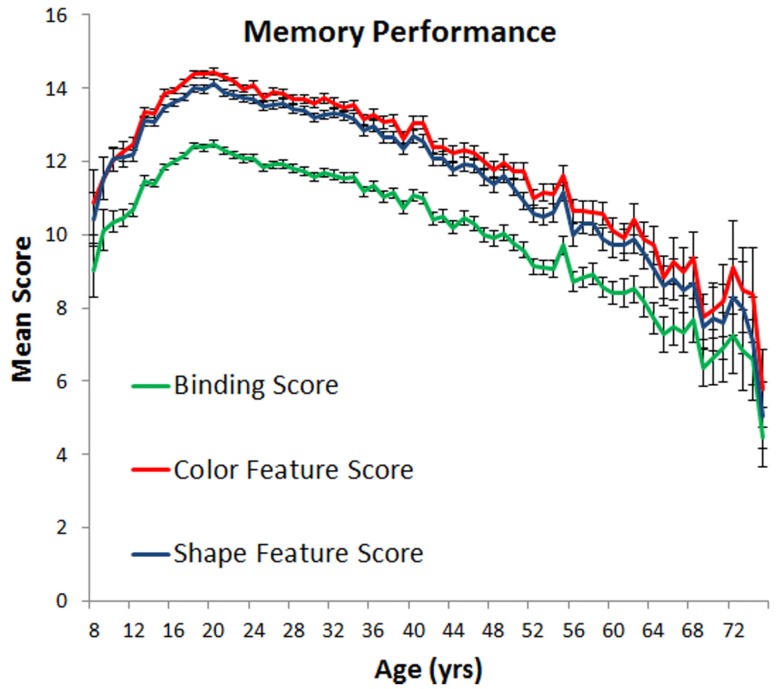
**Mean number of objects remembered (with standard error) as a function of age**.

The results of a one-way ANOVA indicated that object memory varied as a function of age, as measured on a year-by-year basis [*F*(67, 55,752) = 43.4, *p* < 0.0001, $\eta_{p}^{2}=0.05$]. Two clear trends emerged (Figure 3 green line): memory improved from age 8 to 20, while from age 21 to 75, a steady linear decline was observed. Performance observed for participants aged 42–55 was statistically indistinguishable from that of the 8 and 9-year-olds, and statistically poorer performance was observed for those participants aged 56 and over. No differences in performance were observed between males and females throughout the entire age range. Although treating education as a covariate showed a direct relationship between VWM ability and education [*F*(1, 55,684) = 31.4, *p* < 0.0001, $\eta_{p}^{2}=0.0001$], it did not completely temper age-related memory decline [*F*(67, 55,684) = 43.9, *p* < 0.0001, $\eta_{p}^{2}=0.05$].

To investigate binding abilities *per se*, we re-scored performance in two ways (see blue and red lines in Figure 3). A *color feature score* considered any response as correct if a color was placed in the correct location irrespective of the correspondingly selected shape. A *shape feature score* considered a response correct if the shape was placed in the correct location irrespective of the paired color. These scores reveal the quality of memory for individual feature-location bindings because a correct response does not require the proper binding of color to shape.

We first examined memory differences in color and shape memory, controlling for education. A main effect of feature score was reliable [*F*(1, 55,685) = 215, *p* < 0.0001, $\eta_{p}^{2}=0.004$]. Color feature scores (*M* = 13.35) were, on average, slightly higher than shape feature scores (*M* = 12.99), a finding that parallels much of the existing lab-based literature. Trends in color and shape memory were similar across age, however [*F*(67, 55,685) = 1.22, *p* = 0.12]. We therefore simplified our remaining analyses by averaging each participant’s color and shape feature scores to create a single *general feature score* which represents an observer’s ability to remember the identities of individual features irrespective of whether those features are assembled correctly.

We contrasted general feature scores with our original scoring system which considered a response to be correct only if the observer reproduced the precise combinations of features (i.e., bindings between color and shape in the correct location). If memory for bindings is differentially affected by age relative to memory for features, then the effects of age on trends in the two scoring systems should likewise differ. We considered these interactions for both children (age 8–19) and adults (age 20–75). For children, age affected feature memory and binding memory differently [*F*(11, 15,601) = 2.48, *p* < 0.01, $\eta_{p}^{2}=0.002$]. Although quadratic trends were noted, we used linear regression models to estimate the average rate of change in binding scores across the ages tested^3^. On average, feature memory increased at a rate of 0.241 features per 1 year increase in age while binding memory increased at a lesser rate of 0.232 objects per 1 year increase in age. For adults, feature memory and binding memory also had different trajectories [*F*(55, 40,084) = 4.05, *p* < 0.0001, $\eta_{p}^{2}=0.006$]. Linear regression analyses indicated that, on average, feature memory declined at a rate of 0.100 features per 1 year increase in age while binding memory declined at a slower rate of 0.095 objects per 1 year increase in age.

**Figure 4:**
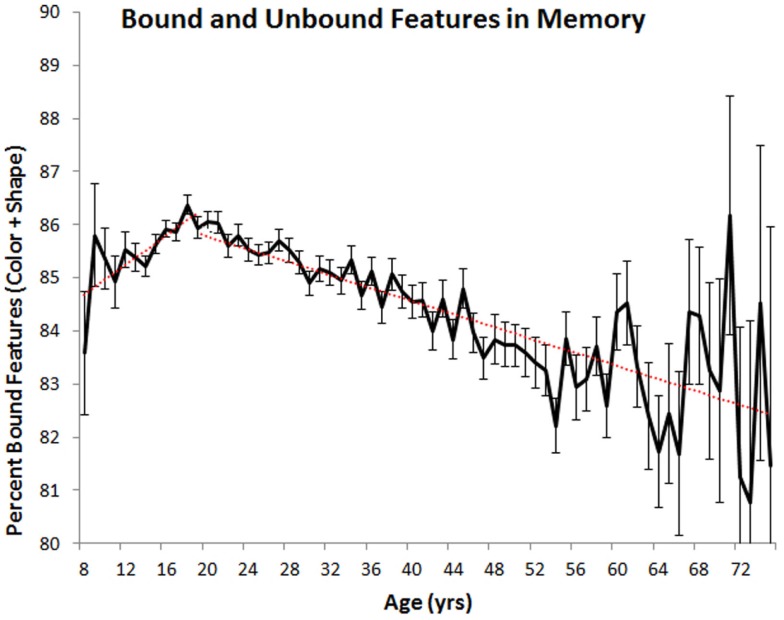
**Mean percentage of remembered colors and shapes that were correctly bound together (with standard error) plotted as a function of age**. Red lines indicate best-fit linear trends.

The differential rates of change in feature and binding memory suggest that the relative proportion of bound and unbound features in VWM may also vary as a function of age(see Figure 4). To assess this possibility, we calculated the number of colors and shapes that were correctly remembered to co-occur (i.e., that were properly bound) and the number of remembered colors and shapes that were not correctly paired in memory (i.e., that were not properly bound). On average, unbound features accounted for 14% of all remembered features in children (ages 8–19) and 16% of all remembered features in adults (ages 20–75). In both cases, the percentage of unbound features varied as a function of year-over-year differences in age [children: *F*(11, 15,601) = 2.733, *p* < 0.05, $\eta_{p}^{2}=0.002$; adults: *F*(55, 40,084) = 7.02, *p* < 0.001, $\eta_{p}^{2}=0.01$]. For children, linear regression analyses indicated that, on average, the percentage of unbound features declined at a rate of 0.14% per 1 year increase in age. For adults, the percentage of unbound features increased at a rate of 0.06% per 1 year increase in age.

To summarize the data, VWM improves throughout adolescence with peak performance observed around age 20. The following decline across adulthood is characterized by a linear trend, with adults in their late 50s and older displaying poorer VWM than 8 and 9 year olds. No difference in performance was observed between men and women and the effects of aging were equivalent across the genders. Higher education was associated with some sparing of visual working memory, but did not completely temper the effects of aging. In terms of feature binding, the proportion of features in memory that are properly bound increases across childhood, with the reverse trend observed in adults.

## Discussion

Using a common metric consistently measured across most of the lifespan on a year-by-year basis, we have shown, like many studies before ours, that VWM abilities change across the lifespan. Regarding children, previous results have shown that performance on VWM tasks improves from birth to at least 11 or 12 years of age. Our study now shows that this improvement continues throughout adolescence, reaching a peak in one’s early twenties whereupon steady declines are observed (see Cornoldi and Vecchi, 2003; Reuter-Lorenz and Sylvester, 2005; Park and Payer, 2006 for reviews). What is more, we have shown that short-term feature memory and short-term binding memory are differentially affected by aging. In childhood, the proportion of properly bound features in memory improves with age, a result which suggests that as children and adolescents get older they are better at remembering feature combinations. In adulthood, the opposite trend is observed, indicating that older adults may have more difficulty remembering bindings. That said, these shifts in binding abilities are very small in comparison to shifts in memory capacity. For example, at age 20, observers remembered, on average, 12.47 of the 20 possible objects they could have viewed. At age 75, they remembered only 4.47 objects, representing a nearly two-thirds drop in capacity. In comparison, 85% of remembered features were properly bound at age 20 compared to 82% for those aged 75. The relative importance of VWM capacity changes are also substantiated by observed effect sizes (see above), with changes in capacity explaining at least five times the variance in performance compared to changes in binding ability.

Aside from the inherent limitations of cross-sectional (as compared to longitudinal) experimental designs, one potential concern with the interpretation of these trends is that the display durations that we used, coupled with a lack of articulatory suppression, might have allowed use of verbal coding of the stimuli, thereby undermining the test as a pure measure of VWM. However, our analyses of the larger dataset from this study (Johnson et al., 2010) have revealed that verbal working memory shows a much slower rate of decline across the adult age range than do measures of VWM. Indeed a test of verbal short-term memory (digit span) showed no age-related decline between the ages of 20 and 65. Therefore, it seems unlikely that the rapid rate of decline in performance across adult aging in the data reported here could be interpreted as reflecting extensive use of verbal codes for colors, shapes, and locations.

Our results, then, are consistent with previous findings (Brockmole et al., 2008) suggesting that age-related decline in short-term color-shape binding memory is driven largely by a decline in capacity for retaining individual features. Short-term color-shape binding shows somewhat different age-related trajectories than individual feature-location binding throughout childhood and appears relatively insensitive to adult aging. This last result is in contrast with the well-established age-related decline in associative learning across the adult lifespan. This possible distinction between temporary feature binding and associative learning is consistent with other findings demonstrating that temporary memory for color-shape bindings may be relatively automatic (e.g., Allen et al., 2006; Gajewski and Brockmole, 2006). The results also suggest that there is considerable merit in further exploring differential changes in binding and feature memory through childhood and adolescence.

## Conflict of Interest Statement

The authors declare that the research was conducted in the absence of any commercial or financial relationships that could be construed as a potential conflict of interest.

## Footnotes

^1^In the literature on visual attention, visual perception, and feature binding, the terms “visual short-term memory” and “visual working memory” tend to be used interchangeably. We view visual short-term memory as comprising a temporary store that is one of a range of functions of visual working memory, which in turn is a set of functions within a broader, multi-component working memory (Logie, 2011), and we use the terms in this way throughout the manuscript. This issue is outside the scope of the present paper and the adopted term “visual working memory” (VWM) is intended to be theoretically neutral here with respect to models of working memory, and how these models account for feature binding. See Logie and van der Meulen (2009) for a review and detailed discussion.

^2^At the time of writing this report, the complete set of tests was available at: http://www.bbc.co.uk/science/humanbody/mind/surveys/memory/flash/one_show_test.shtml. However, the continued availability of these tests is under the control of the BBC, and they might not be available indefinitely.

^3^Quadratic trends are described as follows:

(1) $$Y_{\text{CB}}=4.234-0.018x^{2}+0.771x$$

(2) $$Y_{\text{CF}}=5.114-0.021x^{2}+0.881x$$

(3) $$Y_{\text{AB}}=12.852-0.001x^{2}-0.004x$$

(4) $$Y_{\text{AF}}=14.423-0.001x^{2}+0.012x$$

where *Y* refers to performance, *x* refers to the age of the participant and the subscripts *c*, *a*, *b*, and *f* refer to children, adults, binding score, and feature score, respectively.
